# Intragenic Deletions in *ATP7B* as an Unusual Molecular Genetics Mechanism of Wilson’s Disease Pathogenesis

**DOI:** 10.1371/journal.pone.0168372

**Published:** 2016-12-19

**Authors:** Theodor Todorov, Prahlad Balakrishnan, Alexey Savov, Piotr Socha, Hartmut H. J. Schmidt

**Affiliations:** 1 Department of Transplantation Medicine, University of Muenster, Germany; 2 Institute of Medical Genetics and Genomics, Sir Ganga Ram Hospital, New Delhi, India; 3 National Genetic Laboratory (NGL), Sofia, Bulgaria; 4 Department of Gastroenterology, Hepatology and Immunology, Children’s Memorial Health Institute, Warsaw, Poland; Medizinische Universitat Innsbruck Department fur Kinder- und Jugendheilkunde, AUSTRIA

## Abstract

Wilson’s disease (WD) is an autosomal recessive disorder caused by mutations in the *ATP7B* resulting in copper overload in the liver and brain. Direct sequencing is routinely used to confirm WD diagnosis; however, partial and whole gene deletions in the heterozygous state cannot be detected by exon amplification since the normal allele will mask its presence. The aim of the present work was to search for unusual mutational events in the unexplained WD cases and to provide insight into the mechanisms. Out of 1420 clinically and biochemically confirmed WD samples received between 2000 and 2014 for routine mutation analysis, we were unable to detect mutant alleles in 142 samples, after extensive sequencing analysis. We used selective amplification and MLPA to identify the partial gene deletions and identified three different partial gene deletions in seven different families. All three deletions were fully characterized at the DNA sequence level. We report the first hemizygous case with WD due to intragenic deletion in the *ATP7B* (c.3134_3556+689del). This novel deletion resulted from an excision event mediated by consensus sequences in an AluSq2 repeat element and could be traced to micro homologous end joining (MMEJ). Finally, we determined the prevalence of the three deletions in DNA samples from a multinational group of WD patients. Our results emphasize the need for searching mutant alleles beyond routine methods and highlight that large A*TP7B* deletions are rare, but account for a detectable proportion in some WD patients. Screening for gene aberrations will further improve mutation detection in patients with unidentified *ATP7B* mutations presenting with clinical manifestations of WD.

## Introduction

Wilson’s disease (WD; MIM #27790) is an autosomal recessive inherited disorder of copper metabolism characterized by copper accumulation in the liver and subsequent hepatic and / or neurologic symptoms due to copper toxicity. It is caused by mutations in the *ATP7B* (OMIM *606882), which encodes a transmembrane copper-transporting P-type ATPase. *ATP7B* is located on 13q14.3 and transcribes mRNA of 6655 bp (NM_000053.3) that encodes a protein of 1465 amino acids (NP_000044). A broad spectrum of disease-causing mutations have been reported in *ATP7B*, comprising mostly point mutations and small insertions / deletions (http://www.wilsondisease.med.ualberta.ca/database.asp). The mutation p.H1069Q in exon 14 of *ATP7B* is the most frequently encountered mutation in WD patients of European origin, with large variations in prevalence according to the geographic area and ethnicity [1]^.^ Screening for *ATP7B* mutations is usually performed by direct sequencing. In a substantial number of patients, mutations were identified in only one allele or none. A possibility could be that genome rearrangements and partial gene deletions / duplications of one or more exons are not detected with the current methods used in routine genetic testing. Very few gross rearrangements of *ATP7B* have been described till date. This includes (a) interstitial deletion in the long arm of chromosome 13 that encompasses the *ATP7B* and *RB1* [2] (b) two cases of segmental uniparental isodisomy (UPD13q14.2 to 13q34) found in patients with inheritance of a single parental allele resulting in autozygosity for the mutations p.His1069Gln and p.Arg1319* [3], (c) a homozygous 3039 bp deletion spanning from intron 1 to exon 2 was reported recently [4] and (d) four large *ATP7B* deletions found in four different families have been reported in WD patients; Two deletions with characterized breakpoints were found in the homozygote state, whereas in two other cases the deletion breakpoints were not identified: c.4021+87_4125-2del (ex20del), c.1708-?_1946+?del (ex4_5del) [5,6], c.51+384_1708-953del (ex2*_4del) [7] and c.1544-?_1708-? (ex4del) [8]. The low frequency of partial / full gene deletions in *ATP7B* is surprising given that in the homologous *ATP7A*, intragenic deletions/duplications account for 22% of all mutations resulting in Menkes disease [9]. In a fifteen years period (2000–2014), blood samples from 1420 clinically suspected WD subjects were received in our laboratories for mutation analysis. Notably, in about 10% of clinically and biochemically proven index patients, we were unable to detect mutant alleles even after extensive sequence analysis of promoter, coding region and associated intron-exon boundaries. This study aims to search for partial or whole gene deletions / duplications and large rearrangements in the unexplained WD cases by MLPA assay and other molecular genetics techniques.

Here we present results of our 482 subjects, in which sequencing did not reveal much about the molecular status of WD. We identified three different deletions in 7 different families.

## Materials and Methods

### Subjects

Clinical data as well as biochemical results were evaluated in 2162 WD suspected subjects, who were enrolled for the routine diagnostics study in between 2000–2014 from different countries including Germany, Bulgaria, India and Poland. After thorough initial analysis, we excluded 742 subjects, as they did not confer to the general inclusion criteria (S1 Text) based on clinical and biochemical parameters, adopted from Leipzig 2001 [10]. *ATP7B* was sequenced in the remaining 1420 cases. Of these cases, both the mutations were identified in 1278 (90%). In the remaining 10% (142 subjects), ‘no mutation’ or ‘one mutation’ were found in 70 and 72 cases respectively. Initially only these 142 cases were selected for the study. Later we enrolled patients homozygous for mutations in exons 14, 15, 16, 17, 18, 19 and 20, where both the parents were not available to confirm their true homozygous / segregation state of mutations. Three hundred and forty such WD cases were included.

The inclusion criteria for the present study were ‘no’ or only ‘one’ mutation identified and suspected homozygosity for mutations in the exons 14–20 within the *ATP7B*. Genomic DNA was extracted from peripheral blood samples. Blood was collected after obtaining written informed consent, and all procedures performed were approved by the Institute Ethics Review Board (Ethics Committee of the Medical Association of Westphalia-Lippe and the Medical Faculty of the University of Münster, Germany AZ: 2012-586-f-S). The nomenclature used for nucleotide and amino acid sequence variations was based on established HGVS guidelines at www.hgvs.org/varnomen. The NCBI GenBank *ATP7B* reference sequences NM_000053.3, NG_008806.1 and NP_000044.2 was used.

Two different approaches were utilized to identify the molecular defects. (i) SNP based haplotype analysis to identify the location followed by MLPA analysis and (ii) MLPA analysis to find out the gross deletion. In both the cases sequencing was performed to find out the exact break points.

Extensive analyses were carried out in following cases (a) mutation studies revealed a homozygous p.H1069Q mutation (exon 14) in a Bulgarian female patient, but failed to detect this mutation in the patient’s child as an obligate heterozygote, (b) exons 20 and 21 were not amplified in two patients of Turkish origin (both cases suggested a possible intragenic deletion event(s) of one or more exons in above mentioned *ATP7B* regions) and (c) presence of a deletion of exons 17 to 19 in a patient of Polish origin. The carrier status was determined by testing for the specific mutations in family and /or population by MLPA analysis or direct sequencing of the break points.

### SNP Genotyping

Haplotype analysis was performed using nine intragenic single nucleotide polymorphisms (SNPs) (Table 1) spanning between exon 2 to exon 20. The polymorphisms were analyzed by sequencing the amplified PCR products.

**Table 1:** Intragenic SNPs used for the haplotype analysis and the haplotype of patient and child.

SNPs	Variation	Exon / Intron	Haplotype	
			patient	child
rs1801243	c.1216T>G	2	T/G	T/G
rs1801244	c.1366G>C	3	G/C	G/C
rs1061472	c.2495A>G	10	A/G	A/G
	c.2576-25G>A	IVS10	G/A	G/A
rs732774	c.2855G>A	12	G/A	G/A
	c.2866-90G>T	IVS12	G/T	G/T
	c.2866-105G>A	IVS12	G/A	G/A
rs1801249	c.3419T>C	16	**T/T**	**T/T**
rs2282057	c.3903+6T>C	IVS18	T/C	T/C

### Multiplex Ligation-dependent Probe Amplification

The MLPA kit “SALSA MLPA P098 Wilson disease” was obtained from MRC-Holland, Amsterdam, Netherlands. The kit contains probes for all 21 exons of the *ATP7B*. MLPA was performed according to the manufacturer’s protocol. Reaction products (MLPA) were analyzed on an ABI prism 3730 DNA analyzer using the Gene Scan-500LIZ size standard (from Applied Biosystems). Peak Scanner 1.0 software was used to size the amplicons and to determine the relative peak areas (RPA).

### PCR amplification across the deletions and sequence analysis

The regions of interest in the WD gene were amplified, and sequencing of the PCR products was performed using the BigDye Terminator v3.1 Cycle Sequencing Kit's (Applied Biosystems, Foster City, USA) with an ABI Prism 3730 genetic analyzer (Applied Biosystems). Sequence data was aligned to the reference sequence of the *ATP7B* using Variant Reporter version 1.0 (Applied Biosystems). The identification of the breakpoints enables to design allele specific PCR-assays allowing rapid screening for detected deletions. For analysis of exons 14-16del, 17-19del and exon 20del, the following primers were used: ATP7B14XF 5′ TGTGACTATGGAAGCCCCTC and ATP7BX17R 5′ GCCAACTGGTGCTTACTTTTG; ATP7B16YF 5′ GCTGTTAAAAGGATTGCATGG and ATP7B19YR 5′ CCACTCACTAACCCCAGCAG; and TP7B19ZF 5′ GTGCTGGGAGGGCAATG and ATP7B20ZR 5′ AAGCATGCAGAATGACAAGG respectively.

### Bioinformatic tools

The identification of repetitive sequences was carried out by the CENSOR [11] and Repeat Masker [12] *in silico* tools. In order to detect homologous regions flanking the deletion breakpoints, these were compared using Align Sequences Nucleotide BLAST [13]. For each breakpoint, the neighbouring 300 nucleotides were used to identify homologous / consensus sequences.

## Results

A mutation detection rate of 90% was achieved in our WD cohort of 1420 patients by sequencing. The remaining 142 WD cases along with the 340 cases having homozygous mutations were studied for gross deletions / duplication; we were able to identify three different partial *ATP7B* deletions (S2 Text) in seven different families. Two of them were homozygous for a previously reported partial gene deletion of exon 20 and three patients were compound heterozygous for the same mutation. One patient had intragenic deletions in exons 17–18 and part of exon 19 in heterozygous state, and another was hemizygous for the mutation p.H1069Q in exon14, with a large novel deletion encompassing the same locus (part of exon 14 to intron 16) on the other allele of the *ATP7B*, (as appeared homozygous for p.H1069Q by sequencing earlier).

### SNP Genotyping

**Fig 1:**
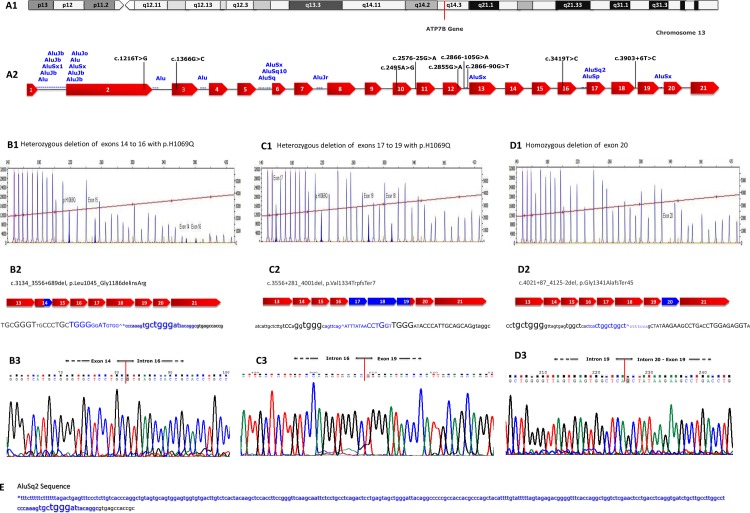
Molecular characterization and breakpoint analysis of large intragenic deletions in the *ATP7B* gene. A1. Schematic illustration of locus q14.3 on chromosome 13. A2. Diagrammatic representation of ATP7B with SNP profile indicated by vertical bars in *ATP7B* gene, the red colored boxes in the genomic sequence represent exons (1–21), Alu content of the *ATP7B* is depicted in blue and SNP in black. B1, C1 and D1. Multiplex ligation-dependent probe amplification gene dosage assay for exons 14 to 16 deletion, exons 17 to 19 and exon 20 respectively. B2, C2 and D2. Schematic representation of the deletion in gene, the blue boxes are the exon regions deleted and the blue nucleotides indicate the deleted fragments. Capital letters represent exon sequences. Asterisk indicates the location of the AluSq2 element in 3′ end of the deleted fragment. B3, C3 and D3. Nucleotide sequence and electropherogram at the breakpoints of the aberrant fragments representing flanking regions of the detected intragenic deletions are shown. E. Enlarged nucleotides highlight consensus sequences near the breakpoints (homologous sequences, elements with internal symmetry and palindromes) as potential hot spots for recombination/deletion events.

Haplotype analysis for suspected hemizygous mutation p.H1069Q in a female patient of Bulgaria was performed using nine intragenic *ATP7B* SNPs with high heterozygosity (Table 1; Fig 1A). Patient and her unaffected child were heterozygous for 8 SNPs and homozygous for exon 16 SNP. On the basis of the order of intragenic SNPs, both share the haplotype T/G-G/C-A/G-G/A-G/A-G/T-G/A-**T/T**-T/C (Table 1). The child inherited the disease haplotype from his mother, and this haplotype lacks C allele of SNP c.3419T>C located in exon 16 suggesting the presence of a mono-allelic *ATP7B* deletion between IVS12 and IVS18 (exons 13–17) encompassing a region of up to 6000bp. MLPA assay was used to confirm and identify the specific region deleted of exon 13–17. Having identified a large deletion of exon 20 in three unrelated families of Turkish origin, we further evaluated the haplotypes to investigate whether the patients were ancestrally related. The deleted alleles share the same haplotype G-C-A-G-A-G-G-C-T for the above mentioned SNP markers.

### MLPA analysis

Additionally MLPA technique was used to analyze 142 unrelated WD patients, which were negative for *ATP7B* mutations on one (n = 72) or both alleles (n = 70). We identified another partial *ATP7B* deletion involving exons 17–19 in a family of Polish origin.

A total of seven alleles showed the deletion of exon 20 and two other alleles provided evidence for the deletions of exons 14–16 and exons 17–19 respectively.

### Characterization of the deletions and estimation of the mutation rate

In order to further characterize the deletions of the *ATP7B*, we performed breakpoint analysis of the detected deletions of exons 14–16, 17–19 and 20 by sequencing. Three site-specific PCR amplification primer sets were designed with each one specifically amplifying one deletion to produce 1549 bp, 605 bp and 590 bp PCR products.

In the Bulgarian family, MLPA as well as the haplotype results suggested a deletion of about 3–4 kb in proband and her son. For both individuals, the ATP7BY14F-17R amplification product was sequenced in both directions. A deletion of 3827 bp was identified, beginning from exon 14 (c.3134) and ending at intron 16 (c.3556+689). This deletion is named c.3134_3556+689del (Fig 1B1 and 1B3). The deletion results in the production of an aberrant protein product of 1080 amino acid residues. In addition, two other deletions of *ATP7B* were also identified. One in a patient of Polish origin, who had c.3556+281_4001del mutation having 3505 bp deletion that includes part of intron 16 to part of exon 19 (Fig 1C1 and 1C3). The mutation is predicted to result in a truncated protein product containing 1341 amino acids (AA) with a net loss of 124 AA. Fine mapping of the second deletion in Turkish proband revealed a loss of 2159 bp including the entire exon 20 (Fig 1D1 and 1D3). The 3′ deletion breakpoint was located in intron 19 (IVS19), and the position of the 5′ breakpoint is located in IVS20. The mutation was described as c.4021+87_4125-2del, p.Gly1341Alafs*45.

We were unable to detect the mutations c.3134_3556+689del and c.3556+281_4001del using multiplex PCR among all tested WD patients including 54 from Bulgaria and 65 from Poland where these mutations were originally identified. The third partial *ATP7B* deletion c.4021+87_4125-2del was observed in a total of 3 out of 72 unrelated Turkish WD families.

We identified five patients with one or more exon(s) deleted, being compound heterozygous. Four of them carried one of the previously observed point mutations (p.H1069Q or p.A874V) located in different exons than the deleted region in the other allele, and one patient carried a mutation (p.H1069Q) in the other allele of the deleted region. The segregation was confirmed by investigating the available parental samples in all these cases. Hence, excluding the possibility of a *de novo* deletion in the present study.

### Bioinformatic analysis

We identified 20 Alu repeat elements, located in 8 of 20 introns, of ATP7B (Fig 1A1 & 1E). Bioinformatics analysis of the deleted regions revealed that the 5′- breakpoint of mutation c.3134_3556+689del lies within an AluSq repeat located in the intron 16 of the *ATP7B* (Fig 1B2). Alignment of the 293 bp AluSq repeat of the consensus Alu sequence showed sequence identity to be 85.3% (250 of 293 nucleotides). Notably, a TGGG sequence located in Alu repeat with both 100% nucleotide identity and orientation in the same direction flanking two of the deletions on both sides (del 14–16 and del 17–19) and the third on one side (Fig 1B2, 1C2 & 1D2), suggesting that the TGGG motif-containing fragments might have mediated the deletion process. Sequences homologous to the immunoglobulin switch region (TGGGG) and the human deletion hotspot consensus hexa nucleotide TGRRKM (R = A or G, K = G or T and M = A or C) [14] is apparent and was found in all *ATP7B* intragenic deletions. Additionally, a deletion hotspot sequence CCTG [14] was found next to the breakpoints of all three deletions (Fig 1B2, 1C2 & 1D2).

## Discussion

Mutations usually linked to WD are identified by sequencing analysis, a method which is unable to detect large gene deletions and rearrangements. Many studies based on sequence analysis of different populations throughout the world have shown a mutation detection rate in the *ATP7B* to be about 80–95%. More than 250 SNPs and 800 mutations in *ATP7B* that result in WD phenotype have been identified (data derived from publications, data banks including our in-house database); variants are partly registered in the WD mutation database and Single Nucleotide Polymorphism Database (dbSNP) [15]. In our large cohort of 1420 patients with WD, the mutation detection rate is 90%. Some of the missing 10% might have been due to major rearrangements. Using MLPA and selective amplification we were able to identify three different partial gene deletions in seven patients with WD. All three deletions were fully characterized at the DNA sequence level.

First aberration is a novel 3827 bp deletion found in a patient hemizygous for mutation p.H1069Q, in whom initially homozygosity was predicted by direct sequencing. In the present case, we were unable to detect the p.H1069Q variant in the patient’s child. Subsequently, suspected deletion in the region of exon 14 was indicated using informative single nucleotide polymorphisms in the flanking exons / introns. The presence of heterozygous allelic variants in exon 2, 3, 10, IVS10, 12, IVS12 and IVS18, but one homozygous polymorphism in exon 16, suggested that a possible deletion between exon 13 and exon 18 has occurred. Deletion of three exons (14–16) was confirmed by MLPA assay. Using a newly designed combination of primers we were able to identify both breakpoints located within exon 14 and 1197 bp upstream of the 3' end of exon16. *This is the first report of hemizygous missense mutation within ATP7B detected in a WD patient highlighting that sequencing alone might falsely indicate a homozygous mutation*. During the course of literature review, we found only a single case of autosomal recessive hemizygous mutation which resulted in partial gene deletion, and was associated with transient congenital hypothyroidism [16].The second deletion (c.3556+281_4001del) was a novel one and was not observed in the 130 alleles analysed from the Polish cohort, where the mutation was originated. This was also not found in rest of the samples (483) included in the study.

The third deletion, of exon 20 was previously described in homozygous state in two Turkish patients [4]. The same mutation was identified in 7 alleles (5 patients from three unrelated family) in our cohort and all of them were of Turkish origin. Considering the relatively high number of the above mentioned exon-deletion mutation in Turkish subjects (7/166 alleles) and absence in other population groups, we speculated that this mutation could have regional origin. We were unable to detect this deletion among the other index cases including those from the neighbouring patient populations (Greece, Bulgaria, Macedonia, and Iran). Our next objective was, whether this deletion arose independently in the three families or whether it was inherited from a common ancestor? Genotyping of a set of nine SNPs covering *ATP7B* (exons 2–20) suggested that the deletion is located on the same haplotype in these 3 unrelated families, indicating that the patients carry the same haplotype. These results rule out a very old founder effect, but support the idea that the deletion does not often arise independently. This is in contrast to Alu-mediated deletions as we reported here, where genetic events may occur more frequently.

We further analyzed the frequencies of these three observed deletions by performing MLPA screening in 66 heterozygous patients and in 70 patients without any *ATP7B* identified mutations, as well as in 340 WD patients homozygous for 27 different mutations (S1 Table) located in the genomic regions of exons 14–16, 17–19 and 20. This cohort has been established from WD mutation analysis of large patient populations in Europe, Asia, and USA. In our screening test for *ATP7B* large deletions / duplications, we did not identify any abnormalities using the MLPA assay. The MLPA assay has the limitation of not identifying large rearrangements that do not change exon dosage, such as inversion, balanced rearrangements or chromosome rearrangements as well as genomic changes that are outside of the sequences targeted by the MLPA probes.

Overall our cohort in this study consisted of 1420 patients. Based on 7 detected patients with exon deletions, the frequency is approximately 0.49%. In comparison, the estimated frequency of deletions in the ATP7A gene (Menkes disease) in almost the same cohort (2500) of patients is 17% [9].

### Possible mechanism

The cause of gross deletions is often related to repetitive elements interspersed throughout the genome. They are commonly found in introns, 3′ untranslated regions of genes and intergenic genomic regions (IGR). Most abundant in the human genome are Alu elements, about 300 bp long and are classified as short interspersed nuclear elements (SINEs). Alu repeat regions are known to be hot spots for recombination events, and the presence of these elements is likely to be the primary cause of genomic rearrangements. Recent studies have reported that Alu-elements result in increased frequency of deletions in human diseases including WD [4,17]. It is also well known that gross deletions can occur during repair mechanism involving ligation of double strand breaks (DSB's) in DNA by non-homologous end-joining recombination (NHEJR), microhomology-mediated replication-dependent recombination (MMRDR) (5–25 bp) and homologous recombination (HR) [18,19]^.^ To investigate developmental mechanism of the detected exon rearrangements, we screened the sequence of *ATP7B* for consensus sequences that are known to be associated with large deletions. *In silico* analysis identified that the new deletion c.3134_c.3556+689; p.Leu1045_Gly1186delinsArg is flanked by an AluSq2 repeat sequence at the 3′ breakpoint side, supporting a mutation mechanism between similar sequences. Short sequence homology between the breakpoint regions may have played a role in the deletion process. The fact that the 3′ breakpoint of the deletion was found to coincide with the AluSq2 element, and more specifically, within a 6 bp overlap of perfectly homologous sequence near their 5′ ends, provides strong evidence for Alu-mediated microhomologous end joining (MMEJ) as pathomechanism (Fig 1E). Bioinformatic analysis revealed that the breakpoint sequences for the exons 17-19del (c.3556+281_4001del, p.Val1334Trpfs*7) show considerable similarity and are also homologous to the breakpoint sequences of exons 14-16del. Furthermore, same extensive homology was found close to the 3′ breakpoint of the exon 20 deletion and is suggestive of a possible role in the mechanism of mutagenesis (Fig 1B). In addition, a TGGAGAGGT element with internal symmetry flank the opposite site of the exon 20 deletion, and forms a TGGAGAG motif identical to the breakpoint consensus sequence encompassing the antithrombin III deletion 14. The junction of the exon 20 deletion doesn’t show high degree of homology between the regions up and downstream of the breakpoints (Fig 1D2) and implies that this deletion was probably caused by non-homologous end-joining (NHEJ). Neither a proximal nor distal breakpoint region of this mutation (c.4021+87_4125-2del), including a recognized full-length repetitive element, was revealed.

Our results reveal that partial gene deletions in *ATP7B* represent causative mutations in some of the uncharacterized WD alleles and MLPA is a suitable and efficient method for identifying such gene alterations. Therefore, each homozygous patient should be considered as potentially hemizygous and the homozygous state has to be confirmed by analyzing both parents / and children (if available) of the WD patient. Absence of the mutation in both parents / patient’s children could indicate the presence of an allelic loss, gross deletion or uniparental isodisomy. However, if methods such as sequencing of genomic DNA, fragment analysis of microsatellite markers and MLPA fail to detect the defective alleles, other factors may contribute to disease. These factors include the impairment of the promoter, polyadenylation regions, and cryptic splice sites lying deep in the intronic regions, defects in the enhancers, silencers, insulators or other regulatory elements of DNA. Furthermore, WD can also be explained by other candidate genes or by factors which may have environmental impact on disease pathogenesis.

In conclusion, we have presented here the results and the application of different methods for the detection of alterations in the exon copy number within the *ATP7B*. Our data highlights that MLPA is a powerful tool to screen for intragenic deletions in WD patients in whom previous analyses of the *ATP7B* failed to identify one or both mutations. By this approach, we were able to reveal 3 different deletions of exons 14–16, 17–19 and exon 20 in 7 patients, two of them have not been reported before. The seven detected alleles with exon 20 deletion among patients with Turkish ethnic origin account for 4% of all *ATP7B* mutant alleles analyzed. The detection of the exon 14–16 deletion emphasizes the need for searching mutations beyond routine methods and that even underlying mutational mechanisms such as Alu mediated deletion processes can be established. This indicates that exonic deletions of *ATP7B* might not be an exceptionally rare cause of WD in some population groups. Thus, the application of MLPA, selection of informative SNPs and identification of deletion breakpoints, in addition to sequencing of genomic DNA is important for improving the mutation detection rate in *ATP7B* patients which is the basis for predictive testing of at-risk relatives.

## Supporting Information

S1 Table Other mutations observed between exon 14 and exon 20. (DOCX)

S1 Text Inclusion / exclusion criteria, Clinical Manifestations and laboratory parameters. (DOCX)

S2 Text Details of the ATP7B gene and Intragenic deletions identified in this study. (DOCX)
