# Supplementary material for: Intragenic Deletions in ATP7B as an Unusual Molecular Genetics Mechanism of Wilson’s Disease Pathogenesis
Source: PLoS One. 2016 Dec 19;11(12):e0168372. doi: 10.1371/journal.pone.0168372 (PMC5167361; doi:10.1371/journal.pone.0168372)
Supplement: S1 Table — (DOCX) [file pone.0168372.s001.docx]

**S1 Table. Other mutations observed between exon 14 and exon 20**

| Exons | Mutations |
| --- | --- |
| Exon 14 | c.3147delC, p.Thr1050fs; c.3182G>A, p.Gly1061Glu; c.3188C>T, p.Ala1063Val; c.3207C>A, p.H1069Q; |
| Exon 15 | c.3266G>A, p.Gly1089Glu; c.3301G>A, p.Gly1101Arg; c.3373_3377delinsTCT, p.His1126ProfsTer3; c.3402delC, p.Ala1135fs* 4 |
| Exon 16 | c.3451C>T, p.Arg1151Cys; c.3458G>A, p.Trp1153Ter; c.3472_3482del11, p.Gly1158fs; c.3556G>T, p.Gly1186Cys |
| Exon 17 | c.3557-2A>G, IVS16-2A>G (IVS16), c.3694A>C, p.Thr1232Pro; c.3649_3654del6, p.Val1217_Leu1218del; c.3664G>A, p.Asp1222Asn |
| Exon 18 | c.3731delT, p.Leu1244Rfs, c.3734C>T, p.Pro1245Leu, c.3742A>C, p.Lys1248Gln, c.3809A>G, p.Asn1270Ser; c.3818C>T, p.Pro1273Leu; c.3895C>T, p.Leu1299Phe, c.3904-2A>G, IVS18-2A>G (IVS18) |
| Exon 19 | c.3955C>T, p.Arg1319Ter; c.3979C>G, p.Leu1327Val; c.3994A>G, p.Asn1332Asp |
| Exon 20 | c.4022G>A, p.Gly1341Asp |
