# Supplementary material for: Intragenic Deletions in ATP7B as an Unusual Molecular Genetics Mechanism of Wilson’s Disease Pathogenesis
Source: PLoS One. 2016 Dec 19;11(12):e0168372. doi: 10.1371/journal.pone.0168372 (PMC5167361; doi:10.1371/journal.pone.0168372)
Supplement: S1 Text — (DOCX) [file pone.0168372.s002.docx]

**S1 Text. Inclusion / exclusion criteria, Clinical Manifestations and laboratory parameters**

The general inclusion / exclusion criteria for molecular genetics evaluations are based on the combinations of clinical signs, symptoms and laboratory findings. Suspected patients with a minimum of two common clinical findings and one laboratory parameter and / or with KF ring were included for the initial genetic evaluation.

**Clinical Manifestations, Ophthalmic evaluation and laboratory parameters**

Wilson disease manifestations may vary from chronic liver disease to fulminant liver failure, progressive neurological disorder without hepatic manifestation, isolated acute hemolysis and even psychiatric disturbances.

The diagnosis of Wilson disease is based on the clinical manifestations and laboratory findings. Careful diagnostic evaluation of an individual with isolated elevated serum transaminase, chronic active hepatitis of non viral origin, unexplained psychiatric symptoms, basal ganglia abnormalities on neuroimaging, Kayser-Fleischer corneal rings (KF rings) and in the laboratory by abnormal levels of serum ceruloplasmin (Cp) (low), urinary copper (high) and liver copper (high) can make the disease visible.

**Hepatic manifestations**

Recurrent jaundice, autoimmune hepatitis, fulminant hepatic failure, chronic liver disease, fatty liver and hemolytic anemia with acute or chronic hemolysis are the common hepatic manifestations. Some common and rare hepatic manifestations are listed below

| **Incidental hepatic manifestations** |
| --- |
| Hepatitis, Acute hepatitis, Chronic hepatitis, Recurrent jaundice, Hepatosplenomegaly, Splenomegaly,  Oedema, Ascites, Cirrhosis, Portal hyper tension, Bleeding from gastric varices, Spider angiomas, Hypoalbuminemia, Coagulation defects, Hepatic failure |

Scheinberg and Sternlieb, 1984; Dobyns *et al.,* 1979; Stremmel *et a*l*.,* 1991; Cox and Roberts, 1998; Kalra *et al.,* 2000; Brewer, 2001; Pandit *et al.,* 2002; Ferenci, 2004

**Neurological manifestations**

Neurological manifestations are diverse and include tremors (Movement disorder like Parkinsonism), dysarthria, focal, segmental and oromandibular dystonia, disphonia, choreoathetosis, hypokinesia, dysarthria, rigidity, bradykinesia and ataxia. Some common and rare neurological manifestations are listed below

| **Incidental neurological manifestations** |
| --- |
| Dystonia, Dysarthria, Tremors, Writing difficulties, Ataxic gait, Hyper salivation, Nervousness, Headache, Mask like face, Dizziness, Convulsions, In-coordination etc. |

Scheinberg and Sternlieb, 1984; Dobyns et al., 1979; Stremmel et al., 1991; Cox and Roberts, 1998; Kalra et al., 2000; Brewer, 2001; Pandit et al., 2002

**Psychiatric manifestations**

Psychiatric manifestations include marked impairment in cognition, abnormal behaviour, personality changes, depression and schizophrenia. Some common and rare psychiatric manifestations are mentioned below

| **Incidental psychiatric problems and manifestations** |
| --- |
| Loss of ability to focus mentally on tasks, Cognitive impairment, Loss of control of emotions, Anxiety, Aggression, Antisocial personality, Temper tantrums, Bouts of crying, Depression, Exhibitionism, Insomnia, Hallucinations, Delusions, Catatonia etc |

Dening and Berrios, 1989; Scheinberg and Sternlieb, 1984; Goldstein et al., 1968; Walker, 1969; Lishman, 1987; Adams and Foley, 1953; Brewer, 2001

**Other manifestations**

The other system manifestations of Wilson disease include hematological, ocular, renal, skeletal, cardiac, endocrinological and muscular.

| **Other Common and rare manifestations** |
| --- |
| **Ocular**  KF ring, Sunflower cataract, Jerky oscillatory movement, Night blindness, Pallor of discs, Xerophthalmia etc.  **Hematological**  Hemolytic anemia, Thrombocytopenia, Cholelithiasis etc.  **Cardiac**  Cardiomyopathy, Congestive heart failure, Conduction abnormalities etc.  **Others**  Hypoparathyroidism, Amenorrhea, Testicular atrophy, Rhabdomyolysis, Arthritis, Rigidity, Arthralgia etc. |

Kayser, 1902; Loudianos and Gitlin, 2002; Saito, 1987; Walshe and Yealland, 1992; Rosenfield et al., 1978; Gitlin, 1998; Bearn et al., 1957

**Diagnostic testing**

**Serum ceruloplasmin**

In Wilson disease patients, the ability of copper to incorporate with apoceruloplasmin to form ceruloplasmin becomes impaired and the production of serum ceruloplasmin is decreased (less than 20 mg/dl)

**24 Hours Urine copper**

If collected in a proper collecting vessel without any copper contamination and loss of urine, 24 hours urine measurement is one of the valuable tests for the diagnosis of Wilson disease. The normal value for 24 hours urinary copper is <50 μg/day (Normal: 20-50 μg/day). In Wilson disease, the value may be >100 μg/day.

**Hepatic copper**

Hepatic copper value of dry liver after liver biopsy is a useful method to evaluate the amount of copper deposited and the severity of the disease. The normal levels are 20 to 50 μg/g dry weight. If the value goes beyond 250 μg/g dry weight, there is a high suspicion of Wilson disease in patients.

**Kayser Fleischer rings**

KF rings are often found in neurologic Wilson disease patients. KF rings represent the deposition of copper in cornea‘s Descemet‘s membrane.

**Routine tests for diagnosis of Wilson disease**

| Test | Typical finding | False negative | False positive |
| --- | --- | --- | --- |
| Serum ceruloplasmin | Decreased | Normal levels in patients with marked hepatic inflammation  Overestimation by immunologic assay | Low levels in: malabsorption  Aceruloplasminemia liver insufficiency heterozygotes |
| 24 hours urinary copper | >100 μg/day | Normal:  Incorrect collection  Children without liver disease | Increased:  Hepatocellular necrosis  Contamination |
| Serum ― free copper | >10 μg/dL | Normal if ceruloplasmin overestimated by immunologic assay |  |
| Hepatic copper | >250 μg/g dry weight | Due to regional variation  In patients with active liver disease, In patients with regenerative nodules | Cholestatic syndromes,  Alcoholic hepatitis |
| KF rings by slit lamp | Present | In up to 40% of patients with hepatic Wilson disease,  In most asymptomatic siblings | Primary biliary cirrhosis |

**Reference**

- Adams RD, Foley (1953) The neurological disorder associated with liver diesease. ARNMD Proceedings 32:198-237.
- Bearn AG, Yu TF, Gutman AB (1957) Renal function in Wilson's disease. J Clin Invest 36:1107-1114
- Brewer GJ Wilson's disease (2001): A clinician's guide to recognition, diagnosis, and management, "London : Kluwer academic publishers,"
- Cox, D.W. and Roberts, E.A. (1998). Wilson disease. In Feldman, M., Schlarschmidt, B.F. and Sleisenger, M.H. (eds), Sleisenger and Fordtran‘s Gastrointestinal and Liver Disease. W.B. Saunders, Philadelphia, PA, 1104–1111.
- Dening TR, Berrios GE (1989) Wilson‘s disease: clinical groups in 400 cases. Acta Neurol Scand. 80:527-534.
- Dobyns WB, Goldstein NP, Gordon H (1979). Clinical spectrum of Wilson‘s disease (hepatolenticular degeneration). Mayo clin Proc 54: 35-42.
- Ferenci P. (2004) Review article: diagnosis and current therapy of Wilson‘s disease. Aliment Pharmacol threr 19: 157-165.
- Gitlin N (1998) Wilson‘s disease: the scourge of copper. J Hepatol 28:734-739.
- Goldstein NP, Ewert JC, Randall RV, Gross JB (1968) Psychiatric aspects of Wilson's disease (hepatolenticular degeneration): results of psychometric tests during long-term therapy. Am J Psychiatry 124:1555-1561
- Kalra V, Khurana D, Mittal R (2000) Wilson's disease-early onset and lessons from a pediatric cohort in India.Indian Pediatr 37:595-601.
- Kayser B (1902) Ueber einen Fall fon angeborener grünlicher Verfärbung der Kornea. Klin Monatsbl Augenheilkd.;40:22-25
- Lishman W A. (1987) Organic Psychiatry. 2nd Ed.. Blackwell. p563-567
- Loudianos G, Gitlin JD (2000) Wilson's disease. Semin Liver Dis 20:353-364
- Pandit A, Bavdekar A, Bhave S (2002) Wilson's disease. Indian J Pediatr 69:785-791
- Rosenfield N, Grand RJ, Watkins JB, Ballantine TV, Levey RH (1978) Cholelithiasis and Wilson disease. J Pediatr 92:210-213
- Saito T (1987) Presenting symptoms and natural history of Wilson disease. Eur J Pediatr 146:261-265
- Scheinberg IH, Sternlieb I. Wilson‘s disease. In: Smith LH Jr., Ed. Major Problems in Internal Medicine. Philadelphia: W.B. Saunders Company, 1984; 23
- Stremmel W, Meyerrose KW, Niederau C, Hefter H, Kreuzpaintner G, Strohmeyer G (1991) Wilson disease: clinical presentation, treatment, and survival. Ann Intern Med 115:720-726
- Walker S, 3rd (1969) The psychiatric presentation of Wilson's disease (hepatolenticular degeneration) with an etiologic explanation. Behavioral neuropsychiatry 1:38-43
- Walshe JM, Yealland M (1992) Wilson's disease: the problem of delayed diagnosis. J Neurol Neurosurg Psychiatry 55:692-696
