# Supplementary material for: Intragenic Deletions in ATP7B as an Unusual Molecular Genetics Mechanism of Wilson’s Disease Pathogenesis
Source: PLoS One. 2016 Dec 19;11(12):e0168372. doi: 10.1371/journal.pone.0168372 (PMC5167361; doi:10.1371/journal.pone.0168372)
Supplement: S2 Text — (DOCX) [file pone.0168372.s003.docx]

**S2 Text. Details of the ATP7B gene and Intragenic deletions identified in this study**

**Gene Summary**

Gene symbol : ATP7B

HGNC ID : 870

Chromosomal location : 13q14.3

Chromosome coordinates : Chromosome 13: 51,932,673-52,011,494 (reverse strand)

GRCh38:CM000675.2

Ensembl Gene : ENSG00000123191.14

Entrez Gene : 540

RefSeq : NM_000053

OMIM : *606882

Protein

Uniprot ID : P35670

Phenotype associated

Conditions : MedGen: C0019202

OMIM: #277900

Orpahanet: ORPHA905

**Deletion 1(Exon 14 – intron 16)**

Chromosome coordinates : Chr13: 51,944,218_51,940,391

HGVS name : c.3134_3556+689del3827

Clinical Significance : Pathogenic

Allele Origin : Maternal

Collection method : Clinical Testing

Affected Status : Yes

Structural Validation method : PCR, MLPA

**Deletion 2 (Intron 16 – exon 19)**

Chromosome coordinates : Chr13: 51,940,800_51,937,295

HGVS name : c.3556+281_4001del3505

Clinical Significance : Pathogenic

Allele Origin : Paternal

Collection method : Clinical Testing

Affected Status : Yes

Structural Validation mehod : PCR, MLPA

**Deletion 3 (Intron 19 – intron 20-exon 21)**

Chromosome coordinates : Chr13: 51,937,188_51,935,029

HGVS name : c.4021+87_4125-2del2159

Clinical Significance : Pathogenic

Allele Origin : Paternal and maternal

Collection method : Clinical Testing

Affected Status : Yes

Structural Validation mehod : PCR, MLPA
